# EPM2A acts as a protective factor in prostate cancer, evidence from a real-world patient cohort

**DOI:** 10.3389/fphar.2022.946637

**Published:** 2022-09-19

**Authors:** Qintao Ge, Jiawei Li, Junyue Tao, Rui Gao, Chen Jin, Jun Zhou, Meng Zhang, Zongyao Hao, Jialin Meng, Chaozhao Liang

**Affiliations:** ^1^ Department of Urology, The First Affiliated Hospital of Anhui Medical University, Hefei, China; ^2^ Institute of Urology, Anhui Medical University, Hefei, China; ^3^ Anhui Province Key Laboratory of Genitourinary Diseases, Anhui Medical University, Hefei, China

**Keywords:** prostate cancer, EMP2A, nomogram, prognosis, chemotherapy, immunotherapy, precision therapy

## Abstract

*EPM2A* encodes a dual specificity phosphatase and has been proven to be a potential biomarker in several cancers but has not been mentioned in prostate cancer (PCA). We investigated the prognostic and therapeutic value of EPM2A in PCA. The TCGA-PRAD cohort was collected to evaluate the differential expression, prognostic value, immunocyte infiltration and drug sensitivity of *EPM2A* in PCA. We constructed a nomogram model to predict the recurrence probability for PCA patients. Immunohistochemistry was used to validate the different transcript levels of *EPM2A* between tumor and normal tissues. A real-world AHMU-PC cohort was employed for validation. The results showed decreased expression of EPM2A in 95.65% of tumor tissues and was related to their prognosis, especially PCA (*p* = 0.008, HR = 0.57, 95% CI: 0.371–0.863). Further multiple analysis by adjusting clinical features revealed that EPM2A acted as an independent prognostic factor (*p* = 0.014, HR = 0.589, 95% CI: 0.386–0.898). Pathway enrichment analysis showed variable signaling activation between high EPM2A expression patients (HEXP) and low EPM2A expression patients (LEXP). The HEXP group contained higher infiltration of immunocytes than the LEXP group, as well as high levels of PD-1, PD-L1 and PD-L2, while LEXP patients were more sensitive to cisplatin, paclitaxel and bicalutamide therapy. The nomogram containing the EPM2A group, T stage and Gleason score showed a preferable prognostic value (AUC = 0.755; Hosmer‒Lemeshow, *p* = 0.486). In validation, we confirmed the lower transcript level of EPM2A in PCA than in normal tissues (120.5 ± 2.159 vs. 138.3 ± 1.83, *p* = 0.035) and correlated it with the expression level of PD-1 (R = 0.283). Among the 66 patients from the AHMU-PC cohort, we further validated the function of EPM2A in PCA patients. HEXP patients had longer recurrence-free survival times (1207 ± 110 vs. 794.2 ± 97.02, *p* = 0.0063) and favorable prognoses (HR: 0.417, 95% CI: 0.195–0.894, *p* = 0.0245). Collectively, we identified the prognostic value of EPM2A in PCa *via* a bioinformatics method. Patients with higher EPM2A may be more sensitive to immunotherapy, and patients with lower EPM2A were more suitable for bicalutamide, cisplatin and paclitaxel therapy.

## Introduction

Prostate cancer (PCA) is among the most common malignancies in males, with approximately 1.6 million one-sets in 2015, and the number is increasing annually (Fitzmaurice et al., 2017). There are many risk factors strongly associated with the incidence of PCA, including age, race and PCA family history. Patients diagnosed with PCA younger than 40 are rarely reported, while the incidence rate increases dramatically after 55 years of age (Ferlay et al., 2015). PCA is also notable for the high variation incidence across racial and ethnic groups. In the United States, the highest incidence rate was observed in African Americans, which was threefold higher than that in Caucasians (Teo et al., 2019). Genome-wide association studies (GWAS) have validated that hereditary heterogeneity plays an essential role in the carcinogenesis of PCA. As a result of heritable genetic and epigenetic alterations, the clinical features, pathological parameters and molecular characteristics are substantially diverse (Alizadeh et al., 2015). A threefold difference was estimated in the incidence rate of PCA between patients with or without a family history of PCA (Howlader et al., 2016). While most PCA run an indolent course without any danger to survival, some will still develop advanced or metastatic disease. With 366,000 deaths and 6.3 million disability-adjusted life-years recorded in 2015, PCA accounts for the fifth highest reason for cancer-related mortality globally (Howlader et al., 2017). To define high-risk PCA, prostate-specific antigen (PSA), T, N, M classification and Gleason scores (GS) have been used to construct risk stratification schemes (Kattan et al., 1998; D’Amico et al., 1998; Chang et al., 2014). However, early detection and stratification strategies to date are limited in the clinical and pathological parameters of PCA without regard to molecular alterations, and there has not been a concomitant improvement in the survival rate for decades. Therefore, a shift toward novel molecular biomarkers from clinicopathological features is essential.

*EPM2A* was first studied for its connection with Lafora disease (LD) (Serratosa et al., 1999; Minassian et al., 2000). It consists of four exons with 130,000 base pairs and is located on chromosome 6q24 (Minassian et al., 1998). *EPM2A* is a highly variable gene, and a total of 43 different gene changes have been cataloged in the Lafora Gene Mutation Database, including homozygous missense, nonsense, frameshift and deletions, all located within its coding region (Ianzano et al., 2005). According to prior studies, the *EPM2A* gene encodes a type of dual specificity phosphatase called laforin, which harbors an amino-terminal carbohydrate binding module and a carboxy-terminal dual specificity phosphatase domain. Laforin is located in the endoplasmic and cytoplasmic rough reticulum and is ubiquitously expressed in all tissues, especially the liver, skeletal muscle and heart (Ganesh et al., 2000; Alonso et al., 2004; Gentry et al., 2013). Although the exact biological functions of laforin remain unclear, its functions in glycogen regulation, protecting cells from ER stress conditions, suppressing cytotoxicity production, autophagy regulation and tumor suppression have been proposed (Worby et al., 2006; Garyali et al., 2009; Vernia et al., 2009; Gentry et al., 2013).

The connection between tumors and *EPM2A* gene expression has attracted much attention. *EPM2A* is depressed in most human lymphomas and acts as a tumor suppressor in immunocompromised hosts by dephosphorylating the essential component of the Wnt signaling pathway, GSK-3β (Wang et al., 2006). As a regulator of glycogen metabolism, EPM2A also contributes to the protection of energy deprivation-induced apoptosis (Wang et al., 2008). This evidence suggests that *EPM2A* gene expression has diagnostic potential in early tumor detection, can reflect the progression trend and may become a novel therapeutic target. However, present studies are limited to several cancers and mainly focus on the molecular mechanisms, while few studies have focused on the association between the expression level and clinical features of PCA. In this study, we found that EPM2A expression was reduced and significantly associated with PCA prognosis. We explored EPM2A as an independent protective factor by univariate and multivariate analyses. Meanwhile, we investigated the antitumour mechanisms of EPM2A based on enrichment analysis, evaluated its predictive value in response to immunotherapy, androgen deprivation therapy (ADT) and chemotherapy, and established a nomogram risk model to provide guidance for quantifying risk stratification.

## Materials and methods

### Raw data collection

In this study, two PCA cohorts were enrolled. The TCGA-PRAD cohort was derived from the GDC platform (https://portal.gdc.cancer.gov/) and contained 488 patients with both gene expression profiles and clinical information. The real-world AHMU-PC cohort obtained from our former works contained both the gene expression profile and corresponding clinical data of 69 patients (Meng et al., 2021). Sixty-nine patients were recruited from those who underwent prostatectomy from 2021 to 2019 in our hospital. Three patients were excluded due to the lack of GS, and 66 patients were eventually enrolled. The TCGA-PRAD cohort was used as the training cohort, and AHMU-PC was used as the validation cohort. All of the expression data were further annotated by the GECODE27 annotation, and log2 (x + 1) transformation was performed. The expression values were expressed as fragments per kilobase per million values (FPKM). All patients were divided into two different groups based on the median value of EPM2A expression. The clinicopathological features of the patients in the two enrolled cohorts are shown in Table 1. We also examined the expression patterns of EPM2A across cancers. The pancancer gene expression profile was downloaded from UCSC Xena (https://xenabrowser.net/datapages/?dataset=GDC-PANCAN.htseq_fpkm-uq.tsv&host=https%3A%2F%2Fgdc.xenahubs.net&removeHub=https%3A%2F%2Fxena.treehouse.gi.ucsc.edu%3A443) and utilized to validate the expression difference of EPM2A among 23 cancers. In addition, biochemical recurrence (BCR), defined as PSA levels greater than 0.2 ng ml−1 measured twice after radical prostatectomy 6–13 weeks, was used as the clinical endpoint. In addition, the evaluation of EPM2A impact on overall survival (OS) and progression-free survival (PFS) was performed on the online GPIEA2 (http://gepia2.cancer-pku.cn/).

**TABLE 1:** Basic clinical features of samples enrolled.

	TCGA-PRAD (*n* = 488)	AHMU-PC (*n* = 66)	Total (*n* = 554)
Status			
Live	396 (81.1%)	37 (56.1%)	433 (78.2%)
Dead	92 (18.9%)	29 (43.9%)	121 (21.8%)
Recurrence-free time			
Mean(standard deviation), months	31.7 (24.8)	32.6 (20.5)	31.8 (24.3)
Median (min, max), months	25.9 (0.8, 164.7)	28.2 (1.6, 79)	26.9 (0.8, 164.7)
Age			
Mean (standard deviation), years	61 (6.8)	69.3 (8.5)	62 (7.5)
Median (min, max), years	61 (41,78)	71 (49,85)	62 (41,85)
T Stage			
T2	187 (38.3%)	53 (80.3%)	240 (43.3%)
T3	291 (59.6%)	11 (16.7%)	302 (54.5%)
T4	10 (2.0%)	2 (3.0%)	12 (2.2%)
Gleason			
6	44 (9.0%)	16 (24.2%)	60 (10.8%)
7	243 (49.8%)	21 (31.8%)	264 (47.7%)
8	61 (12.5%)	13 (19.7%)	74 (13.4%)
9	137 (28.1%)	14 (21.2%)	151 (27.3%)
10	3 (0.6%)	2 (3.0%)	5 (0.9%)

### Enrichment analyses

The differentially expressed genes (DEGs) between the high EPM2A expression level and low EPM2A expression level groups were calculated by the “limma” package. The absolute value of fold-change > 0.4 and *P* adjusted value < 0.01 were set as cutoff values to filter the DEGs. Gene Ontology (GO), Kyoto Encyclopedia of Genes and Genomes (KEGG) and HALLMARK enrichment analyses were conducted with the R package “org.Hs.eg.db” (Ashburner et al., 2000) and further annotated *via* the R package “clusterProfiler” (Yu et al., 2012). These pathways were illustrated by the R package “enrichplot” (Zhang et al., 2019).

### Evaluation of immunocyte infiltration

To reveal the immunocyte infiltration status in the tumor microenvironment (TME), we collected 28 immunocyte signatures from a prior study (Yoshihara et al., 2013). The normalized enrichment score (NES) for the 28 signatures in each PCA patient was calculated *via* a single sample gene set enrichment algorithm (ssGSEA) *via* the R package “GSEA”, and the results are shown in a heatmap (Subramanian et al., 2007).

### Efficacy prediction of response to androgen deprivation therapy, chemotherapy and immunotherapy

Based on the TCGA-PRAD cohort, we investigated the ADT and chemotherapy prediction efficacy of the novel risk model using the comDrugen module in the R package “MOVICS” with the half maximal inhibitory concentration (IC50) as the index (Lu et al., 2020). In addition, we investigated the correlation between EPM2A expression and the expression of PD-1, PD-L1 and PD-L2 in PCA. The results were visualized *via* box plots.

### Nomogram for predicting survival

We established a reliable nomogram risk prediction model with three risk parameters that have been proven to be independent protective or risk factors in prior works to predict the 3-year and 5-year recurrence rates. Receiver operating characteristic (ROC) analysis was performed to calculate the temporal AUC and C-index, and the calibration was analyzed at 3 and 5 years to evaluate the discrimination ability.

### Immunohistochemical staining for EPM2A and PD-1 and additional validation in a real-world cohort

We employed immunohistochemistry (IHC) staining to investigate the differential expression of EPM2A (anti-EPM2A antibody: Cat. Ab129110, Abcam Inc., MA, United States) between tumor and normal tissues and the PD-1 expression level (anti-PD-1 antibody: Cat. Ab52587, Abcam Inc., MA, United States). A tissue microarray of the PCA cohort (HPro-Ade045PG-01) consisting of three normal tissues and 41 tumor tissues purchased from OUTDO IVD, Shanghai, China, was used in this study. The detailed procedures of IHC have been proposed in prior studies (Yin et al., 2019). Multiple semiquantitative grading systems were employed to evaluate the expression status of EPM2A and PD-1 in IHC staining sections, including positive stained region scores and immunostaining intensity scores. The positively stained region scores were set as four degrees: 0 (negative), 1 (1%–10%), 2 (11%–50%), 3 (51%–80%) and 4 (>80% positive area). The intensity scores were also set as four degrees: 0 (no staining), 1 (weak), 2 (mild), and 3 (strong intensity). Based on the data in the AHMU-PC cohort, we conducted survival analysis to confirm the different RFS rates among the EPM2A groups. For the established nomogram risk model, we calculated nomogram points for each sample in the AHMU-PC cohort and separated them into high and low nomogram point groups with their median values. Kaplan–Meier curves were drawn to illustrate different RFS rates among the nomogram point groups. To evaluate the effectiveness and accuracy of the nomogram, ROC analysis was generated, and the area under the curve (AUC) and C-index were calculated.

### Statistical analyses

All statistical analyses were performed *via* R version 4.0.2. For continuous data, the Wilcoxon rank-sum test was applied for comparisons if the values were abnormally distributed, or Student’s *t*-test was used. For categorical data, the chi-square test and Fisher’s exact test were used. Different recurrence-free survival (RFS) rates were shown based on a log-rank test and Cox proportional hazards regression for hazard ratios and their 95% confidence intervals (CI) and visualized *via* Kaplan‒Meier curves. Univariate and multifactorial Cox regression analyses were performed to explore the independent prognostic values of EPM2A after adjusting for clinical features. A two-tailed *p* value < 0.05 was considered significant.

## Result

### EPM2A is a suppressor across cancers

**FIGURE 1:**
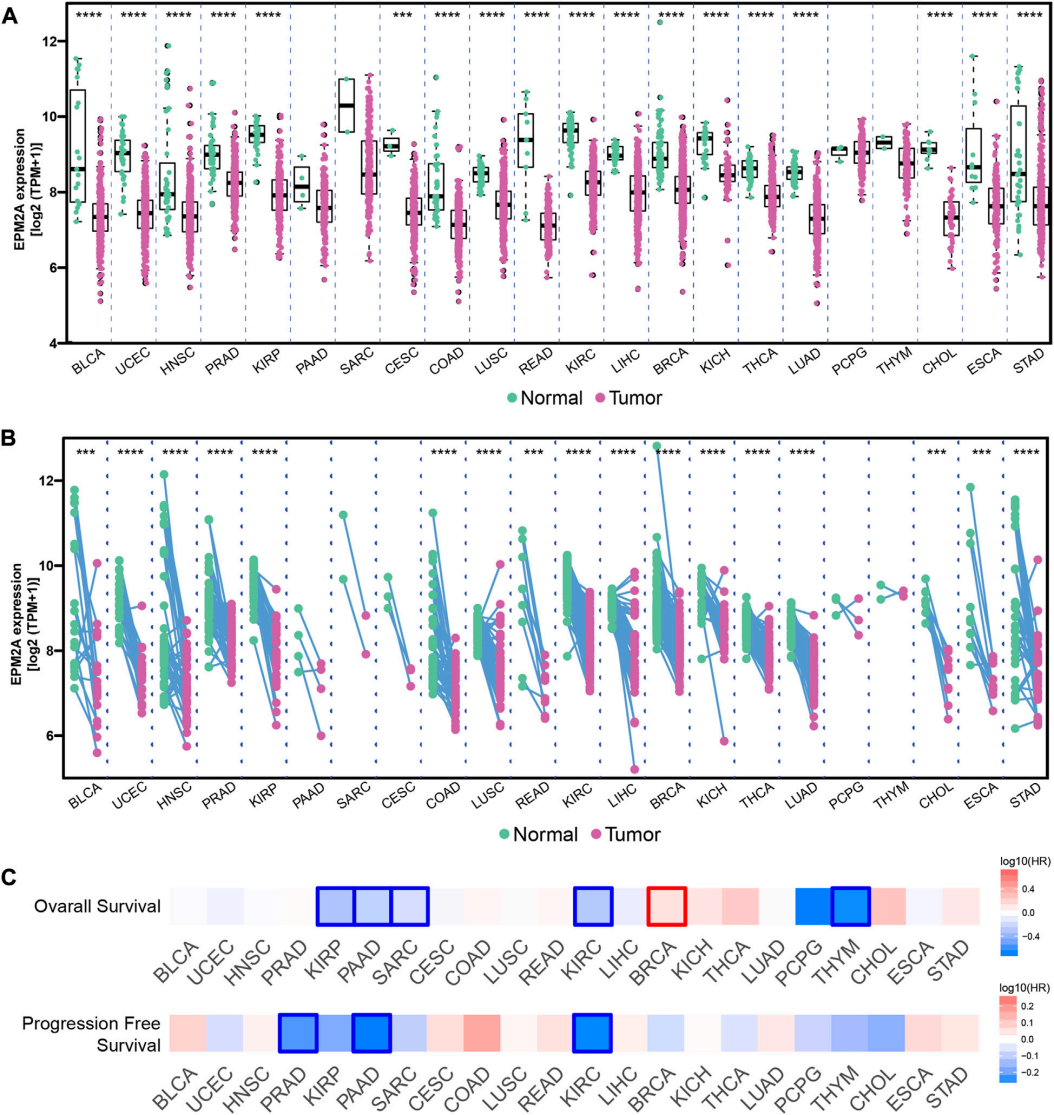
Prognostic value of EPM2A in pancancer. **(A)** Different expression of EPM2A between 22 tumor tissues and adjacent tissues. **(B)** Different expression of EPM2A between 22 tumor tissues and adjacent tissues based on the data from TCGA project. **(C)** Prognostic significance of EPM2A to OS and PFS in pancancer.

In this study, to investigate the prognostic significance of EPM2A, we first compared the expression pattern among tumor and adjacent tissues in 22 tumors. Decreased expression levels of EPM2A were observed in most tumors, such as PCA and bladder cancer, compared to normal tissues (Figure 1A, *p* < 0.05, 21/22). Further validation of the deceased expression of EPM2A in tumor vs. paired adjacent tissues was performed with pancancer data from TCGA project (Figure 1B, *p* < 0.05). The pancancer expression pattern of EPM2A prompted us to explore its prognostic value. We set OS and PFS as clinical outcome indicators to evaluate the predictive potential of EPM2A across cancers. As the results demonstrated, patients with high EPM2A expression had a significantly longer OS and PFS (Figure 1C, *p* < 0.05) in most tumors, especially in PCA.

### The EPM2A expression level is positively correlated with good prognosis

**FIGURE 2:**
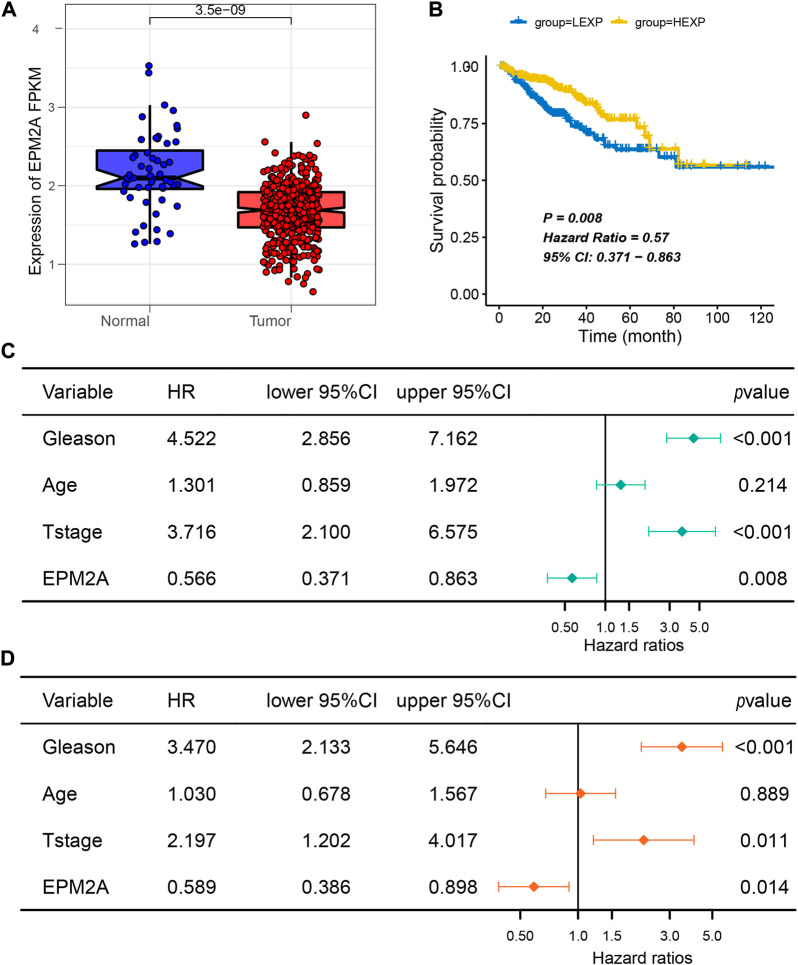
Prognostic value of EPM2A in PCA. **(A)** Comparison of EPM2A expression among prostate tumor and corresponding normal tissues. **(B)** Kaplan–Meier curves for EPM2A groups. **(C,D)** Univariate regression analysis and multivariate regression analysis showed the independent prognostic factors of PCA.

Further research therefore focused on the role of EPM2A in PCA. Likewise, lower expression of EPM2A was observed in PCA tissues (Figure 2A, *p* = 3.5e-09). To investigate the association between EPM2A and RFS in PCA patients, survival analysis was performed, and Kaplan‒Meier curves revealed that high expression of EPM2A was positively associated with longer RFS (Figure 2B, *p* = 0.008, HR = 0.57, 95% CI: 0.371–0.863). Based on this finding, more efforts have been made to delve into the connection between EPM2A and more clinical features. Univariate regression analysis identified GS (*p* < 0.001, HR = 4.522, 95% CI: 2.865–7.162) and T stage (*p* < 0.001, HR = 3.72, 95% CI: 2.1–6.575) as risk factors for PCA, while EPM2A was significantly associated with good prognosis (*p* = 0.008, HR = 0.566, 95% CI: 0.371–0.863, Figure 2C). After eliminating other impacts in multivariate regression analysis, EPM2A (*p* = 0.014, HR = 0.589, 95% CI: 0.386–0.898) was validated as an independent protective factor, and GS (*p* < 0.001, HR = 3.47, 95% CI: 2.133–5.646) and T stage (*p* = 0.011, HR = 2.197, 95% CI: 1.202–4.017) were identified as independent risk factors for PCA (Figure 2D). In addition, we investigated the clinicopathological feature distributions between the high and low EPM2A subgroups, and the results showed that patients with low EPM2A expression levels had higher Gleason scores and tumor stages. The low-EPM2A group contained more patients with T stage > 2 than the high-EPM2A group in both the TCGA-PRAD (165 vs. 136) and AHMU-PC (4 vs. 9) cohorts. Although the Gleason score exhibited no statistically significant difference between the EPM2A groups, low-EPM2A patients tended to have a higher Gleason score, and the difference might be more pronounced as the sample size increased (Supplementary Tables S1, S2). After preliminary research, EPM2A was confirmed to be a biomarker in PCA, and the expression level was positively correlated with good prognosis.

### Enrichment analysis revealed the antitumour mechanisms

**FIGURE 3:**
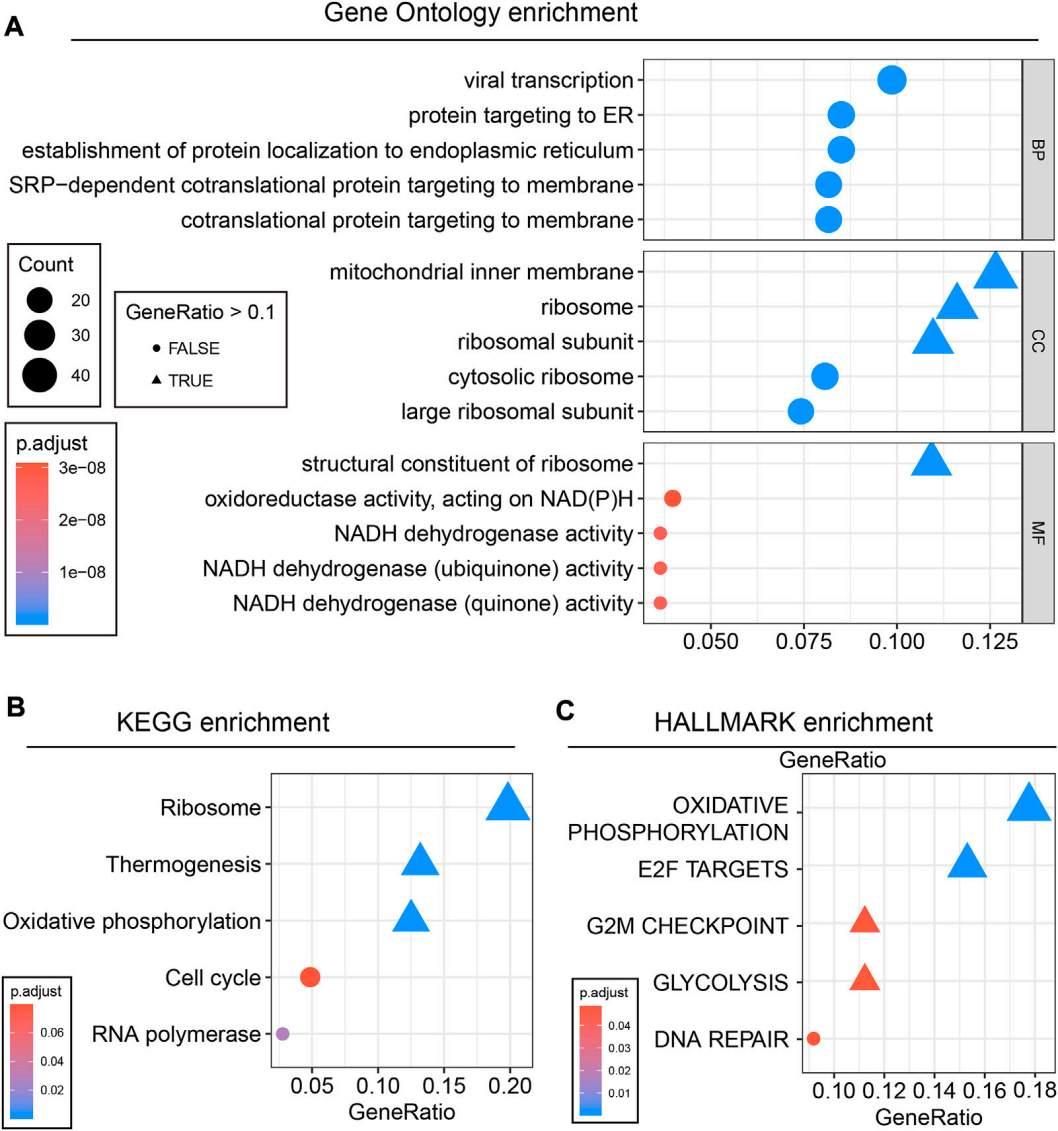
Enrichment analysis revealed the tumor-suppressive mechanism of EPM2A in PCA. **(A)** 376 DEGs were enriched in different pathways, and the top five GO terms are showed. **(B)** The top five KEGG terms. **(C)** The top five HALLMARK terms.

Next, the cancer antitumour mechanisms of EPM2A were further investigated with enrichment analysis. A total of 376 DEGs between the high and low EPM2A subgroups were identified with a preset cutoff value and further annotated in different biological progress items. We found the most significantly altered pathways, and the top five GO, KEGG and HALLMARK pathways are illustrated (Figures 3A–C). After integrating the results with a previous study, we considered that EPM2A can impact PCA development through protein targeting and establishment to the endoplasmic reticulum, cotranslational protein to the membrane, participation in ribosome structural constituents, oxidoreductase activity, oxidative phosphorylation and glycolysis (Vernia et al., 2009). Discovery of the antitumour mechanism not only convinced the prognostic role but also provided a potential therapeutic target for drug development.

### EPM2A is a novel predictor of immunocyte infiltration and the PD-1/PD-L1/PD-L2 expression landscape

**FIGURE 4:**
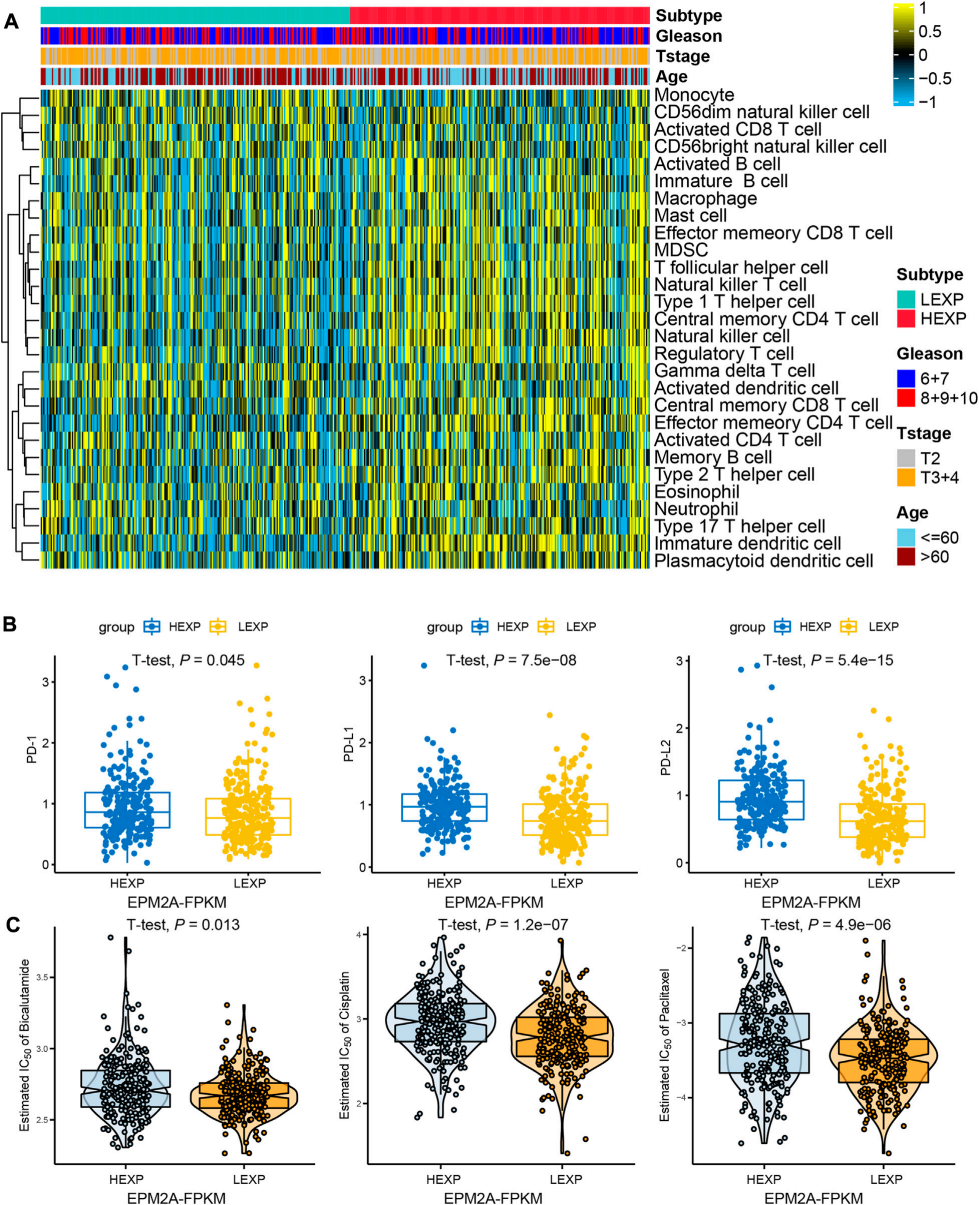
Immunocyte infiltration landscape and therapy prediction. **(A)** Heatmap showed the comparisons of immunocyte infiltration status between EPM2A groups. **(B)** Differential expression of PD-1, PD-L1 and PD-L2 among the two newly defined groups. **(C)** Comparisons of the estimated IC50 of bicalutamide, cisplatin and paclitaxel in the two newly defined groups.

Compared to other solid malignancies, current immunotherapy has particular limitations in PCA. It has been reported that the prostate cancer TME forms an unsuitable niche for the antitumor function of infiltrated immunocytes and leads to the limited efficacy of immunotherapy for PCA patients (Madan and Gulley, 2019). Precision immunotherapy for PCA is thus of great significance for promoting therapeutic efficacy. In this study, higher infiltration of immunocytes was observed in the high EPM2A groups. This difference was particularly prominent in activated B cells, immature B cells, macrophages, mast cells, effector memory CD8 T cells, T follicular cells, natural killer cells, type 1 helper cells, regulator cells, central memory T cells, effector memory CD4 T cells, memory B cells, type 2 T helper cells, type 17 helper cells and immature dendritic cell clusters (Figure 4A). Interestingly, high T cell infiltration, especially CD8 T cells, was considered to contribute to the increased efficacy of immune checkpoint inhibitors (Bruni et al., 2020; Meng et al., 2021). Meanwhile, we found higher PD-1, PD-L1 and PD-L2 expression in the high EPM2A expression subgroup (Figure 4B, *p* < 0.05) than in the low EPM2A expression subgroup. All these findings strongly suggest the latent predictive value of EPM2A for PCA immunotherapy.

### EPM2A is a novel predictor of chemotherapy and androgen deprivation therapy

The molecular classification system has also been proven to be an efficacy tool to predict the clinical response to chemotherapy in many cancers (McConkey et al., 2016; Kamoun et al., 2020). Further studies focused on whether EPM2A can be a predictor in the response of PCA to ADT and chemotherapy. As shown, a higher IC50 was observed in the high EPM2A subgroup than in the low EPM2A subgroup in chemotherapy classes, suggesting that patients with low EPM2A expression are more suitable for the treatment of cisplatin (*p* = 1.2e-07) and paclitaxel (*p* = 4.9e-06). Meanwhile, we examined whether EPM2A can be a predictor for ADT. Likewise, the comparison of IC50 values between the EPM2A groups suggested that high EPM2A levels were related to a poor response to bicalutamide (Figure 4C, *p* = 0.013).

### Nomogram risk prediction model construction

**FIGURE 5:**
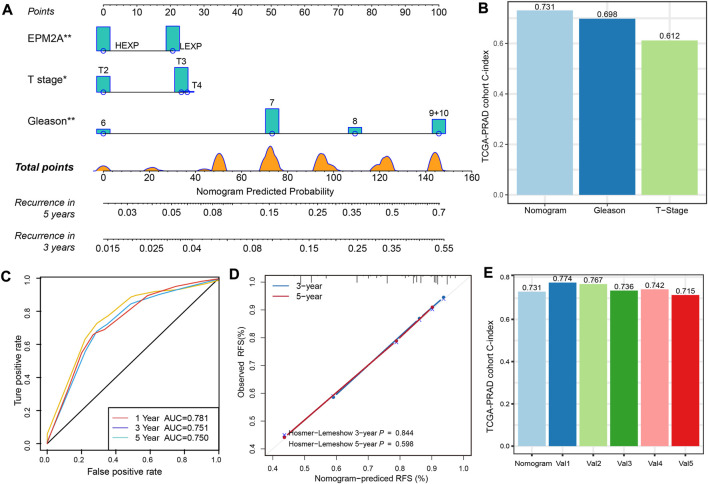
A nomogram for prognostic prediction of an individual patient. **(A)** Prognostic prediction nomogram with EPM2A, Gleason score and T stage. For a given patient, scores are plotted on corresponding scales, and the value is from the vertical lines to the top points scale. The sum of the scores for each predictor represent the total score and is plotted on the bottom scale showing the predicted probability of 3-year and 5-year recurrence rates. **(B)** C-index of the nomogram, T stage and Gleason score based on the data from TCGA-PRAD cohort. **(C)** Temporal ROC curves for the nomogram and the value of AUC at 1, 3 and 5 years. **(D)** Calibration curves and Hosmer-Lemeshow test at 3 and 5 years for the nomogram. **(E)** Internal validation of the nomogram following a cross validation analysis, and Val1 to Val5 represented the five independent operations.

We constructed a nomogram risk prediction model with three factors, including EPM2A expression level, T stage and GS, which have been validated as independent prognostic factors. For a given patient, the sum of the scores for each predictor represents the total score corresponding to the probability of 3-year and 5-year recurrence (Figure 5A). Moreover, we found that GS was the most important weight factor. Interestingly, the weight of EPM2A was almost the same as that of the T stage. To further analyze the incremental predictive value of EPM2A added to the model, we calculated and compared the C-index for the nomogram, T stage and GS. The values of the C-index for the nomogram, T stage and GS in the TCGA-PRAD cohort were 0.731, 0.698 and 0.612, respectively, which indicated an increase in the C-index after combining EPM2A with T stage and GS. Therefore, the combination of EPM2A with T stage and GS potentially improved the discrimination ability of the predictive model (Figure 5B). ROC analysis was performed to verify the discrimination of our model, and the AUC values at 1, 3 and 5 years were 0.781, 0.751 and 0.75, respectively, indicating a relatively stable prediction performance within 5 years (Figure 5C). Furthermore, the predictive value of the nomogram was assessed by calibration analysis, and the results from Hosmer‒Lemeshow analysis showed that the nomogram-predicted probability was highly consistent with the actual recurrence probability, and the 3-year and 5-year *p* values were 0.844 and 0.596, respectively (Figure 5D). We randomly selected 250 samples from the TCGA-PRAD cohort to establish a nomogram for internal validation, and the C-index was calculated to evaluate the predictive power. The above process was repeated 5 times independently, and the corresponding C-index was also calculated and compared. As Figure 5E shows, the C-index of the original nomogram and five independent operations were 0.731, 0.774, 0.767, 0.736, 0.742 and 0.715, indicating a stable predictive performance of the clinical model.

### Extra validation of EPM2A prognostic significance

**FIGURE 6:**
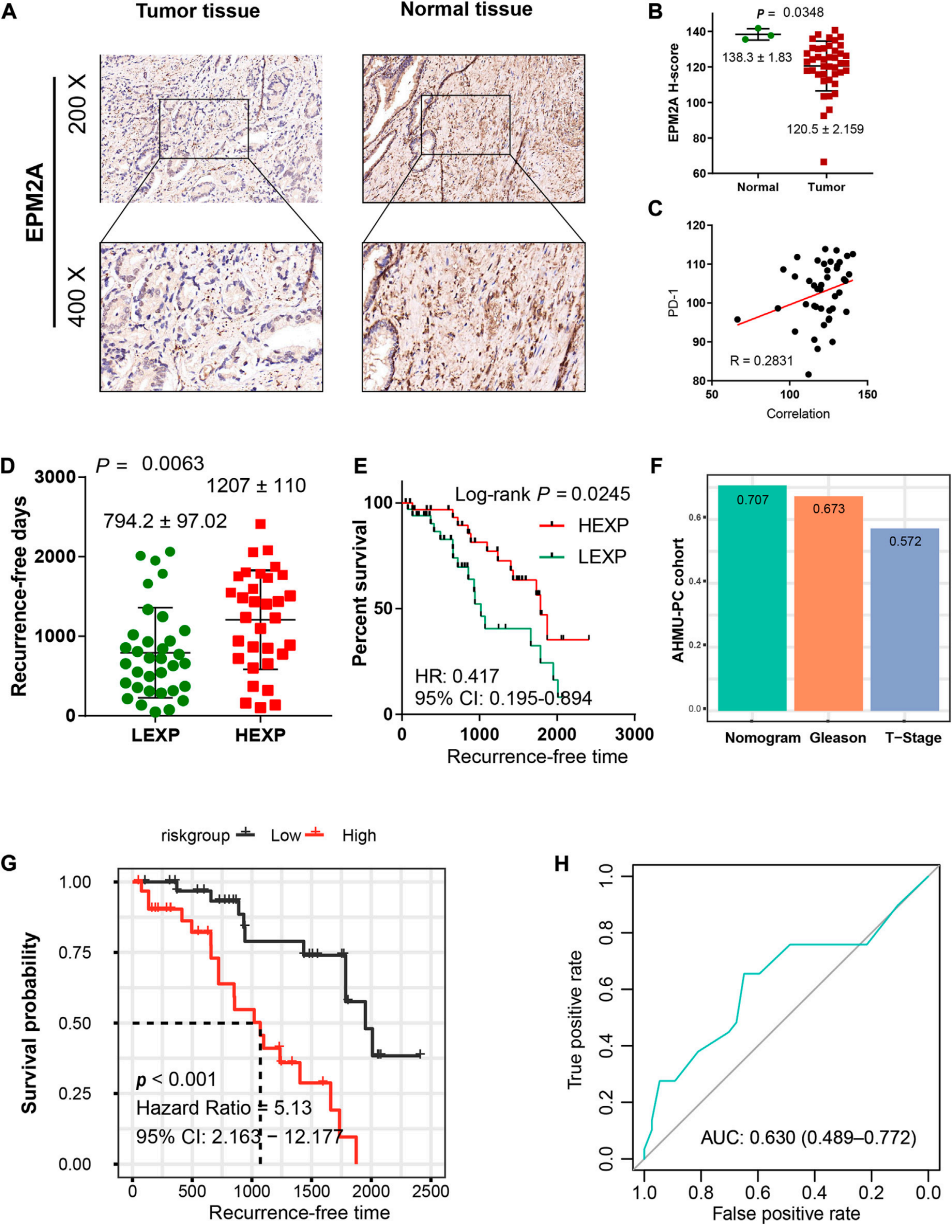
Validation of the predictive value in the AHMU-PC cohort. **(A)** IHC illustrated the different EPM2A expression levels between PCA tumor and normal tissues. **(B)** Comparison of EPM2A H-scores between tumor and normal tissues. **(C)** The positive linear correlation between EPM2A expression level and PD-1 expression level. **(D)** The comparison of recurrence-free days between high and low EPM2A expression groups **(E)** Kaplan–Meier curves showed significantly different recurrence-free times between the two groups. **(F)** C-index of the nomogram, T stage and Gleason score based on the data from AHMU-PC cohort. **(G)** K-M curves for the nomogram based on the data from AHMU-PC cohort, high and low nomogram point groups were divided *via* the median value. **(H)** ROC curve analysis was performed to assess the reliability of the established nomogram.

To confirm the decreased expression of EPM2A in PCA, IHC was employed, and a lower H-score was observed in the tumor slides, which is consistent with the result that EPM2A was downregulated in PCA tissues (Figures 6A,B, 138.3 ± 1.83 vs. 120.5 ± 2.159, *p* = 0.0348). With IHC, we also found that the high expression level of EPM2A was positively linearly correlated with PD-1 expression in PCA cells (R = 0.28) (Figure 6C). In addition, we enrolled transcription and corresponding clinical data from a real-world AHMU-PC cohort (*n* = 66) to further test the prognostic value of EPM2A in PCA. Patients in the high EPM2A group showed longer RFS with a 2.25-fold HR than those in the low EPM2A group (794.2 ± 97.02 vs. 1207 ± 110, *p* = 0.0063), with a 95% CI from 0.195 to 0.894 (log-rank *p* = 0.0245) (Figures 6D,E). Similarly, we calculated and compared the C-index for the nomogram, T stage and GS based on the AHMU-PC cohort, and the C-index values were 0.707, 0.673 and 0.572, respectively. The higher C-index of the complete predictive model confirmed that EPM2A was able to improve the discriminative ability of the model (Figure 6F). To evaluate the predictive power of the complete predictive model, we calculated recurrence probability for patients in the AHMU-PC cohort using the nomogram and divided them into high and low subgroups with the mean value, and the results showed that patients with high recurrence scores indeed had significantly shorter RFS than those with low recurrence scores. (Figure 6G, *p* < 0.001, HR = 5.13, 95% CI = 2.163–12.177). The AUC value of 0.630 (0.489–0.772) indicated that the results were reliable (Figure 6H).

## Discussion

In recent years, PCA has become the most common solid cancer in men, with an increased incidence annually, accounting for 9% of male cancer deaths (Brockman et al., 2015). With widespread PSA screening, the postoperative BCR for PCA is nearly 1/3. In countries where PSA detection is not widely applied, patients are often diagnosed with advanced metastatic PCA and eventually the emergence of metastatic castration-resistant prostate cancer (mCPRC), which is considered a lethal reason for the shorter OS of PCA patients (Schatz and Mian, 2017; Rui et al., 2019). The treatment schemes to date include radical surgery, external beam radiotherapy, ADT, chemotherapy and immunotherapy. However, none of them are satisfactory to all patients, and approximately 27%–53% will develop local recurrence or distant metastasis within 10 years after the operation (Grossfeld et al., 1998; Bott, 2004). PSA monitoring is the main strategy for detecting early PCA recurrence. However, an increase in PSA cannot reflect the full landscape of PCA recurrence, and urologists are therefore double challenged with both avoiding overtreatment and preventing the onset of clinical progression (Artibani et al., 2018). It is urgent to take into account more cancer-specific biomarkers to reflect clinical progression and guide treatment strategies for different individuals.

In this study, we first conducted a pancancer analysis to reveal the differential expression of EPM2A in 22 cancers. Decreased EPM2A was observed in most cancer types, and the decreased expression level was highly related to unfavorable OS and PFS, especially in PCA. Our focus was thus shifted toward the prognostic significance of EPM2A for PCA. Based on TCGA-PRAD cohort, we observed higher EPM2A PCA had a significantly longer OS than low EPM2A PCA, which was validated in univariate Cox regression analysis, and further multivariate Cox regression analysis manifested the independent role of EPM2A to prognosis. Although the mechanisms of how EPM2A impacts the prognosis of PCA remain unclear, protein targeting and establishment to the endoplasmic reticulum, cotranslational proteins to the membrane, participation in ribosome structural constituents, oxidoreductase activity, oxidative phosphorylation and glycolysis were identified as the crucial pathways. Our results are supported by previous research demonstrating the mechanisms of EPM2A in inhibiting tumor development. For instance, the EPM2A transcription product has been found to be the specific phosphatase for GSK-3β, which is a key modulator of several essential signal transduction pathways. Overexpression of EPM2A diphosphates GSK-3β at the Ser9 position (Lohi et al., 2005) and then modulates the pivotal player in the initiation, maintenance and development of tumors by inhibiting the activation of the Wnt signaling pathway (Duchartre et al., 2016).

Immunotherapy has been a promising cancer treatment modality in the last few years. Despite breakthroughs in immune checkpoint inhibitor (ICI) immunotherapy, not all patients have a satisfactory response to it, especially prostate cancer. It is a widely accepted view that ICI-mediated antitumour responses are dependent on the T-cell infiltration status. Tumors with high T-cell infiltration, which are also called “hot tumors”, are considered to have a high response to immunotherapy, while tumors with low T-cell infiltration, which are also called “cold tumors”, are considered to have a poor response to immunotherapy (Liu and Sun, 2021). PCA is on the “cold tumors” spectrum with minimal T-cell infiltrates, indicating a poor response to ICI immunotherapy (Bilusic et al., 2017). However, a large, placebo-controlled, randomized phase III clinical trial suggested that ICI immunotherapy is still a potential treatment strategy for PCA (Beer et al., 2017). Therefore, further insights into how to select sensitive patients receiving immunotherapy are urgently needed. We found significant differences in immunocyte infiltration between patients with high and low EPM2A expression. High EPM2A expression might be a biomarker for high immunocyte infiltration, and further analysis found a positive linear correlation between EPM2A and PD-1 expression. These results strongly indicated the potential predictive value of EPM2A for immunotherapy. More effort would be put in this point. According to prior studies, high-risk PCA was defined to have a positive margin, extraprostatic extension, lymph node involvement, high PSA or high Gleason score after primary therapy. In these patients, ADT played an essential role in shrinking the tumor, reducing margin positivity and preventing BCR (Fang and Zhou, 2019). However, long-term ADT can induce PCA to develop into mCRPC. For these patient groups, chemotherapy was employed. Chemotherapeutic and ADT agents have greatly improved PCA patient OS in recent decades. However, much of the complexity of primary PCA treatment for each patient is rooted in the multifocal nature of the disease (Haffner et al., 2021). New predictive biomarkers for ADT and chemotherapy are considered to have the potential for optimizing therapy by personalizing treatment strategies. Our study provided a successful attempt using the EPM2A molecule to predict therapeutic efficacy for different individuals. We believe it could be a supplemental method for PCA diagnosis and treatment.

Furthermore, we established a nomogram with three covariates for predicting 3-year and 5-year recurrence rates for PCA based on the TCGA-PRAD cohort. Compared to the AJCC staging system or single pathological parameter staging system, our novel risk model is more quantitative and intuitive and is easy for clinicians to use (Amin et al., 2017). More importantly, the novel risk model took molecular features into account, which has been widely believed to be an essential complement to traditional risk stratification schemes, and different risk scores were calculated to reflect the weights of different prognostic factors. To evaluate the discriminative of the novel nomogram, we calculated the C-index for the multivariate model of the T stage and the Gleason score, and the C-index of the complete prognostic model. The higher C-index of the complete predictive value manifested the incremental predictive of EPM2A added to the clinical model. Meanwhile, temporal AUC at 1, 3 and 5 years and Hosmer−Lemeshow *p* value at 3 or years were calculated, as well as an internal validation of the nomogram following a cross validation analysis. Finally, we applied the nomogram risk model to the AMHU-PC cohort to calculate the nomogram point for each patient and divided them into two groups with the median value. Patients in the high nomogram point group showed significantly shorter RFS than those in the low point nomogram point group, proving the stability and reliability of our risk model.

## Conclusion

Taken together, we revealed that EPM2A acts as an independent prognostic factor in PCA and elucidated the antitumour mechanisms. Patients with higher EPM2A expression may be more sensitive in response to immunotherapy but have a poor response to bicalutamide, cisplatin and paclitaxel therapy. Meanwhile, we constructed a nomogram risk model and wish to offer individual clinical endpoint predictions and optimize personalized treatment for each patient. There are still a few limitations to our study. Only 488 samples in the training cohort and 66 samples in the validation cohort were enrolled, and the sample size was small. We confirmed the decreased expression of EPM2A by IHC, and more experiments will be performed to determine the expression pattern in tumor and normal tissues in PCA.

## Data Availability

The original contributions presented in the study are included in the article/Supplementary Material, further inquiries can be directed to the corresponding authors.

## References

[B1] Alizadeh A. A. Aranda V. Bardelli A. Blanpain C. Bock C. Borowski C. (2015). Toward understanding and exploiting tumor heterogeneity. Nat. Med. 21 (8), 846–853. 10.1038/nm.3915 26248267 PMC4785013

[B2] Alonso A. Sasin J. Bottini N. Friedberg I. Friedberg I. Osterman A. (2004). Protein tyrosine phosphatases in the human genome. Cell 117 (6), 699–711. 10.1016/j.cell.2004.05.018 15186772

[B3] Amin M. B. Greene F. L. Edge S. B. Compton C. C. Gershenwald J. E. Brookland R. K. (2017). The Eighth Edition AJCC Cancer Staging Manual: Continuing to build a bridge from a population-based to a more "personalized" approach to cancer staging. Ca. Cancer J. Clin. 67 (2), 93–99. 10.3322/caac.21388 28094848

[B4] Artibani W. Porcaro A. B. De Marco V. Cerruto M. A. Siracusano S. (2018). Management of biochemical recurrence after primary curative treatment for prostate cancer: A review. Urol. Int. 100 (3), 251–262. 10.1159/000481438 29161715

[B5] Ashburner M. Ball C. A. Blake J. A. Botstein D. Butler H. Cherry J. M. (2000). Gene ontology: Tool for the unification of biology. The gene ontology consortium. Nat. Genet. 25 (1), 25–29. 10.1038/75556 10802651 PMC3037419

[B6] Beer T. M. Kwon E. D. Drake C. G. Fizazi K. Logothetis C. Gravis G. (2017). Randomized, double-blind, phase III trial of ipilimumab versus placebo in asymptomatic or minimally symptomatic patients with metastatic chemotherapy-naive castration-resistant prostate cancer. J. Clin. Oncol. 35 (1), 40–47. 10.1200/JCO.2016.69.1584 28034081

[B7] Bilusic M. Madan R. A. Gulley J. L. (2017). Immunotherapy of prostate cancer: Facts and hopes. Clin. Cancer Res. 23 (22), 6764–6770. 10.1158/1078-0432.CCR-17-0019 28663235 PMC5690854

[B8] Bott S. R. (2004). Management of recurrent disease after radical prostatectomy. Prostate Cancer Prostatic Dis. 7 (3), 211–216. 10.1038/sj.pcan.4500732 15278094

[B9] Brockman J. A. Alanee S. Vickers A. J. Scardino P. T. Wood D. P. Kibel A. S. (2015). Nomogram predicting prostate cancer-specific mortality for men with biochemical recurrence after radical prostatectomy. Eur. Urol. 67 (6), 1160–1167. 10.1016/j.eururo.2014.09.019 25301759 PMC4779062

[B10] Bruni D. Angell H. K. Galon J. (2020). The immune contexture and Immunoscore in cancer prognosis and therapeutic efficacy. Nat. Rev. Cancer 20 (11), 662–680. 10.1038/s41568-020-0285-7 32753728

[B11] Chang A. J. Autio K. A. Roach M. 3rd Scher H. I. (2014). High-risk prostate cancer-classification and therapy. Nat. Rev. Clin. Oncol. 11 (6), 308–323. 10.1038/nrclinonc.2014.68 24840073 PMC4508854

[B12] D'Amico A. V. Whittington R. Malkowicz S. B. Schultz D. Blank K. Broderick G. A. (1998). Biochemical outcome after radical prostatectomy, external beam radiation therapy, or interstitial radiation therapy for clinically localized prostate cancer. Jama 280 (11), 969–974. 10.1001/jama.280.11.969 9749478

[B13] Duchartre Y. Kim Y. M. Kahn M. (2016). The Wnt signaling pathway in cancer. Crit. Rev. Oncol. Hematol. 99, 141–149. 10.1016/j.critrevonc.2015.12.005 26775730 PMC5853106

[B14] Fang D. Zhou L. (2019). Androgen deprivation therapy in nonmetastatic prostate cancer patients: Indications, treatment effects, and new predictive biomarkers. Asia. Pac. J. Clin. Oncol. 15 (3), 108–120. 10.1111/ajco.13108 30729683 PMC6850478

[B15] Ferlay J. Soerjomataram I. Dikshit R. Eser S. Mathers C. Rebelo M. (2015). Cancer incidence and mortality worldwide: Sources, methods and major patterns in GLOBOCAN 2012. Int. J. Cancer 136 (5), E359–E386. 10.1002/ijc.29210 25220842

[B16] Fitzmaurice C. Allen C. Barber R. M. Barregard L. Bhutta Z. A. Brenner H. (2017). Global, regional, and national cancer incidence, mortality, years of life lost, years lived with disability, and disability-adjusted life-years for 32 cancer groups, 1990 to 2015: A systematic analysis for the global burden of disease study. JAMA Oncol. 3 (4), 524–548. 10.1001/jamaoncol.2016.5688 27918777 PMC6103527

[B17] Ganesh S. Agarwala K. L. Ueda K. Akagi T. Shoda K. Usui T. (2000). Laforin, defective in the progressive myoclonus epilepsy of Lafora type, is a dual-specificity phosphatase associated with polyribosomes. Hum. Mol. Genet. 9 (15), 2251–2261. 10.1093/oxfordjournals.hmg.a018916 11001928

[B18] Garyali P. Siwach P. Singh P. K. Puri R. Mittal S. Sengupta S. (2009). The malin-laforin complex suppresses the cellular toxicity of misfolded proteins by promoting their degradation through the ubiquitin-proteasome system. Hum. Mol. Genet. 18 (4), 688–700. 10.1093/hmg/ddn398 19036738

[B19] Gentry M. S. Romá-Mateo C. Sanz P. (2013). Laforin, a protein with many faces: Glucan phosphatase, adapter protein, et alii. Febs J. 280 (2), 525–537. 10.1111/j.1742-4658.2012.08549.x 22364389 PMC3371293

[B20] Grossfeld G. D. Stier D. M. Flanders S. C. Henning J. M. Schonfeld W. Warolin K. (1998). Use of second treatment following definitive local therapy for prostate cancer: Data from the caPSURE database. J. Urology 160 (4), 1398–1404. 10.1097/00005392-199810000-00050 9751363

[B21] Haffner M. C. Zwart W. Roudier M. P. True L. D. Nelson W. G. Epstein J. I. (2021). Genomic and phenotypic heterogeneity in prostate cancer. Nat. Rev. Urol. 18 (2), 79–92. 10.1038/s41585-020-00400-w 33328650 PMC7969494

[B22] Howlader N. N. A. Krapcho M. Miller D. Bishop K. Altekruse S. F. Kosary C. L. (2016). SEER cancer statistics review 1975–2013. Bethesda: National Cancer Institute. MD2016 Available at: https://seer.cancer.gov/archive/csr/1975_2013/ .

[B23] Howlader N. N. A. Krapcho M. Miller D. Bishop K. Altekruse S. F. Kosary C. L. (2017). SEER cancer statistics review, 1975–2014. Bethesda, MD: Natl. Cancer Inst. Available at: http://seer.cancer.gov/csr/1975_2014 .

[B24] Ianzano L. Zhang J. Chan E. M. Zhao X. C. Lohi H. Scherer S. W. (2005). Lafora progressive Myoclonus Epilepsy mutation database-EPM2A and NHLRC1 (EPM2B) genes. Hum. Mutat. 26 (4), 397. 10.1002/humu.9376 16134145

[B25] Kamoun A. de Reyniès A. Allory Y. Sjödahl G. Robertson A. G. Seiler R. (2020). A consensus molecular classification of muscle-invasive bladder cancer. Eur. Urol. 77 (4), 420–433. 10.1016/j.eururo.2019.09.006 31563503 PMC7690647

[B26] Kattan M. W. Eastham J. A. Stapleton A. M. Wheeler T. M. Scardino P. T. (1998). A preoperative nomogram for disease recurrence following radical prostatectomy for prostate cancer. J. Natl. Cancer Inst. 90 (10), 766–771. 10.1093/jnci/90.10.766 9605647

[B27] Liu Y. T. Sun Z. J. (2021). Turning cold tumors into hot tumors by improving T-cell infiltration. Theranostics 11 (11), 5365–5386. 10.7150/thno.58390 33859752 PMC8039952

[B28] Lohi H. Ianzano L. Zhao X. C. Chan E. M. Turnbull J. Scherer S. W. (2005). Novel glycogen synthase kinase 3 and ubiquitination pathways in progressive myoclonus epilepsy. Hum. Mol. Genet. 14 (18), 2727–2736. 10.1093/hmg/ddi306 16115820

[B29] Lu X. Meng J. Zhou Y. Jiang L. Yan F. (2020). Movics: an R package for multi-omics integration and visualization in cancer subtyping. Bioinformatics 36, 5539–5541. 10.1093/bioinformatics/btaa1018 33315104

[B30] Madan R. A. Gulley J. L. (2019). Finding an immunologic beachhead in the prostate cancer microenvironment. J. Natl. Cancer Inst. 111 (3), 219–220. 10.1093/jnci/djy145 30321404 PMC6657437

[B31] McConkey D. J. Choi W. Shen Y. Lee I. L. Porten S. Matin S. F. (2016). A prognostic gene expression signature in the molecular classification of chemotherapy-naïve urothelial cancer is predictive of clinical outcomes from neoadjuvant chemotherapy: A phase 2 trial of dose-dense methotrexate, vinblastine, doxorubicin, and cisplatin with bevacizumab in urothelial cancer. Eur. Urol. 69 (5), 855–862. 10.1016/j.eururo.2015.08.034 26343003 PMC4775435

[B32] Meng J. Zhou Y. Lu X. Bian Z. Chen Y. Zhou J. (2021). Immune response drives outcomes in prostate cancer: Implications for immunotherapy. Mol. Oncol. 15 (5), 1358–1375. 10.1002/1878-0261.12887 33338321 PMC8096785

[B33] Minassian B. A. Ianzano L. Delgado-Escueta A. V. Scherer S. W. (2000). Identification of new and common mutations in the EPM2A gene in Lafora disease. Neurology 54 (2), 488–490. 10.1212/wnl.54.2.488 10668720

[B34] Minassian B. A. Lee J. R. Herbrick J. A. Huizenga J. Soder S. Mungall A. J. (1998). Mutations in a gene encoding a novel protein tyrosine phosphatase cause progressive myoclonus epilepsy. Nat. Genet. 20 (2), 171–174. 10.1038/2470 9771710

[B35] Rui X. Shao S. Wang L. Leng J. (2019). Identification of recurrence marker associated with immune infiltration in prostate cancer with radical resection and build prognostic nomogram. BMC Cancer 19 (1), 1179. 10.1186/s12885-019-6391-9 31795990 PMC6892211

[B36] Schatz A. Mian B. M. (2017). Current and emerging trends in prostate cancer immunotherapy. Asian J. Androl. 21 (1), 6–11. 10.4103/aja.aja_52_17 PMC6337946 29176187

[B37] Serratosa J. M. Gómez-Garre P. Gallardo M. E. Anta B. de Bernabé D. B. Lindhout D. (1999). A novel protein tyrosine phosphatase gene is mutated in progressive myoclonus epilepsy of the Lafora type (EPM2). Hum. Mol. Genet. 8 (2), 345–352. 10.1093/hmg/8.2.345 9931343

[B38] Subramanian A. Kuehn H. Gould J. Tamayo P. Mesirov J. P. (2007). GSEA-P: A desktop application for gene set enrichment analysis. Bioinformatics 23 (23), 3251–3253. 10.1093/bioinformatics/btm369 17644558

[B39] Teo M. Y. Rathkopf D. E. Kantoff P. (2019). Treatment of advanced prostate cancer. Annu. Rev. Med. 70, 479–499. 10.1146/annurev-med-051517-011947 30691365 PMC6441973

[B40] Vernia S. Rubio T. Heredia M. Rodríguez de Córdoba S. Sanz P. (2009). Increased endoplasmic reticulum stress and decreased proteasomal function in lafora disease models lacking the phosphatase laforin. PLoS One 4 (6), e5907. 10.1371/journal.pone.0005907 19529779 PMC2692001

[B41] Wang Y. Liu Y. Wu C. McNally B. Liu Y. Zheng P. (2008). Laforin confers cancer resistance to energy deprivation-induced apoptosis. Cancer Res. 68 (11), 4039–4044. 10.1158/0008-5472.CAN-07-6314 18519661 PMC2440919

[B42] Wang Y. Liu Y. Wu C. Zhang H. Zheng X. Zheng Z. (2006). Epm2a suppresses tumor growth in an immunocompromised host by inhibiting Wnt signaling. Cancer Cell 10 (3), 179–190. 10.1016/j.ccr.2006.08.008 16959610

[B43] Worby C. A. Gentry M. S. Dixon J. E. (2006). Laforin, a dual specificity phosphatase that dephosphorylates complex carbohydrates. J. Biol. Chem. 281 (41), 30412–30418. 10.1074/jbc.M606117200 16901901 PMC2774450

[B44] Yin Y. Xu L. Chang Y. Zeng T. Chen X. Wang A. (2019). N-Myc promotes therapeutic resistance development of neuroendocrine prostate cancer by differentially regulating miR-421/ATM pathway. Mol. Cancer 18 (1), 11. 10.1186/s12943-019-0941-2 30657058 PMC6337850

[B45] Yoshihara K. Shahmoradgoli M. Martínez E. Vegesna R. Kim H. Torres-Garcia W. (2013). Inferring tumour purity and stromal and immune cell admixture from expression data. Nat. Commun. 4, 2612. 10.1038/ncomms3612 24113773 PMC3826632

[B46] Yu G. Wang L. G. Han Y. He Q. Y. (2012). clusterProfiler: an R package for comparing biological themes among gene clusters. Omics 16 (5), 284–287. 10.1089/omi.2011.0118 22455463 PMC3339379

[B47] Zhang C. Zheng Y. Li X. Hu X. Qi F. Luo J. (2019). Genome-wide mutation profiling and related risk signature for prognosis of papillary renal cell carcinoma. Ann. Transl. Med. 7 (18), 427. 10.21037/atm.2019.08.113 31700863 PMC6803196

