# Supplementary material for: EPM2A acts as a protective factor in prostate cancer, evidence from a real-world patient cohort
Source: Front Pharmacol. 2022 Sep 19;13:946637. doi: 10.3389/fphar.2022.946637 (PMC9527317; doi:10.3389/fphar.2022.946637)
Supplement: Supplementary file 1 [file DataSheet1.docx]

**Supplementary table1**. Clinicopathological feature distributions between EPM2A groups in TCGA-PRAD cohort.

|  |  | **HEXP (N=240)** | **LEXP (N=248)** | **P-value** |
| --- | --- | --- | --- | --- |
| **Gleason** |  |  |  |  |
| 6 |  | 24 (10.0%) | 20 (8.1%) | 0.536 |
| 7 |  | 124 (51.7%) | 119 (48.0%) |  |
| 8 |  | 30 (12.5%) | 31 (12.5%) |  |
| 9+10 |  | 62 (25.8%) | 78 (31.5%) |  |
| **Age** |  |  |  |  |
| <=65 |  | 167 (69.6%) | 181 (73.0%) | 0.465 |
| >65 |  | 73 (30.4%) | 67 (27.0%) |  |
| **T-stage** |  |  |  |  |
| T2 |  | 104 (43.3%) | 83 (33.5%) | 0.052 |
| T3 |  | 130 (54.2%) | 161 (64.9%) |  |
| T4 |  | 6 (2.5%) | 4 (1.6%) |  |

**Supplementary table2**. Clinicopathological feature distributions between EPM2A groups in AHMU-PC cohort.

|  | **HEXP (N=32)** | **LEXP (N=34)** | **P-value** |
| --- | --- | --- | --- |
| **Gleason** |  |  |  |
| 6 | 8 (25.0%) | 8 (23.5%) | 0.523 |
| 7 | 11 (34.4%) | 10 (29.4%) |  |
| 8 | 6 (18.8%) | 7 (20.6%) |  |
| 9+10 | 7 (21.9%) | 9 (26.5%) |  |
| **Age** |  |  |  |
| <=65 | 9 (28.1%) | 10 (29.4%) | 1 |
| >65 | 23 (71.9%) | 24 (70.6%) |  |
| **T-stage** |  |  |  |
| T2 | 28 (87.5%) | 25 (73.5%) | 0.231 |
| T3 | 4 (12.5%) | 7 (20.6%) |  |
| T4 | 0 (0%) | 2 (5.9%) |  |
